# Exploiting human and mouse transcriptomic data: Identification of circadian genes and pathways influencing health

**DOI:** 10.1002/bies.201400193

**Published:** 2015-03-14

**Authors:** Emma E. Laing, Jonathan D. Johnston, Carla S. Möller‐Levet, Giselda Bucca, Colin P. Smith, Derk‐Jan Dijk, Simon N. Archer

**Affiliations:** ^1^ School of Biosciences and MedicineFaculty of Health and Medical SciencesUniversity of SurreyGuildfordSurreyUK

**Keywords:** bioinformatics, clock gene, gene expression, Reverb, sleep disruption

## Abstract

The power of the application of bioinformatics across multiple publicly available transcriptomic data sets was explored. Using 19 human and mouse circadian transcriptomic data sets, we found that *NR1D1* and *NR1D2* which encode heme‐responsive nuclear receptors are the most rhythmic transcripts across sleep conditions and tissues suggesting that they are at the core of circadian rhythm generation. Analyzes of human transcriptomic data show that a core set of transcripts related to processes including immune function, glucocorticoid signalling, and lipid metabolism is rhythmically expressed independently of the sleep‐wake cycle. We also identify key transcripts associated with transcription and translation that are disrupted by sleep manipulations, and through network analysis identify putative mechanisms underlying the adverse health outcomes associated with sleep disruption, such as diabetes and cancer. Comparative bioinformatics applied to existing and future data sets will be a powerful tool for the identification of core circadian‐ and sleep‐dependent molecules.

**Abbreviations**
GO: gene ontology
TSD: total sleep deprivation
UTR: untranslated region

## Introduction

The advent of high‐resolution high‐throughput technologies has enabled the simultaneous measurement of mRNA levels for all genes within a mammalian system under particular conditions. Several groups interested in mammalian circadian rhythms have now used these technologies to identify genes that are expressed rhythmically during “normal” (i.e. controlled conditions that bear some similarity to habitual conditions) and “perturbed” conditions (e.g. the forced desynchronisation of the sleep‐wake cycle and central clock in the brain, forced wakefulness, removal of the light/dark cycle, manipulations of scheduled feeding times) in species including human and mouse (e.g. 1, 2, 3, 4, 5, 6, 7, 8, 9). Clearly, as each research group performs the most suitable experiment to test their own hypotheses, there are large differences in protocols (perturbations), the type of sample taken (tissue, blood etc.), the resolution of sampling times, the numbers of subjects, technological platforms, and the analytical algorithms used to identify rhythmic signals. This variability across experiments can be a significant obstacle in the comparative analysis of gene specific responses across conditions and/or mammalian systems. However, the variability across experiments can also be exploited to address specific research questions from a slightly different perspective. For example, if we were to identify a set of genes that are expressed rhythmically across multiple experiments from different organisms, experimental set‐ups, laboratories, and statistical analyses, then those genes, by the very nature of their identification, could be classified as “robust rhythmic genes.” While this list of robust genes is likely to be an under‐estimation, due to the cross‐comparison of different data sets, thresholds etc., we speculate that such genes are most likely to be closely associated with the core circadian machinery of mammalian systems.

In this paper we report a comparative analysis of circadian genes (i.e. genes with a circadian expression profile) identified from two human gene expression datasets that comprise four conditions: 40 hours of total sleep deprivation (TSD) following sufficient sleep (∼8.5 hours per night), and 40 hours of TSD following insufficient sleep (∼5.7 hours per night) 7, and sleeping either in phase with melatonin, a marker of the central hypothalamic clock and biological night, or sleeping 12 hours out of phase with melatonin 2. We first identified genes that were circadian in these human in vivo experiments. To further investigate the robustness of these circadian genes, we compared them with genes that were identified as circadian in cultured human bone cells 4, human hair follicles 1, post‐mortem human brain tissue 5, in brain and liver from two mouse sleep deprivation studies 3, 6, and multiple tissue expression datasets from mice 8, 9. This approach identified a core set of mainly human‐blood specific rhythmic genes across sleep conditions, some core circadian clock genes that were generally rhythmic across all samples, and several core clock genes that appear to be differentially rhythmic between human or mouse samples. Further bioinformatic analysis determined: which manipulations of the sleep‐wake cycle impacted most upon circadian rhythmicity; the genes affected by disrupted sleep; and the underlying processes with potential links to associated adverse health conditions.

## Congruency of human rhythmic gene expression signals

A multitude of biochemical and physiological pathways exhibit daily changes in function. These rhythms are driven in part by environmental cycles and behavioral cycles, as well as by endogenous, self‐sustaining circadian oscillators found throughout the various organs and tissues of the body, including blood cells. The predominant molecular model of the clock describes a set of interlocking transcriptional‐translational feedback loops. In the core loop, CLOCK (and in some tissues NPAS2) and BMAL1 proteins stimulate transcription of three *PERIOD* (*PER*) and two *CRYPTOCHROME* (*CRY*) genes. The resulting PER and CRY proteins then form complexes that translocate into the nucleus and inhibit transcription of their own genes by interacting with CLOCK and BMAL1. Post‐translational modification, for example by phosphorylation of PER by CSNK1ε and CSNK1δ, regulates the stability and activity of clock proteins and is a major determinant of the periodicity of the molecular feedback loop 10.

The core molecular loop is thought to interlink with numerous secondary loops that enable it to interact with other aspects of cell physiology and metabolism. One such “secondary loop” involves the nuclear receptors NR1D1 (REVERBα) and NR1D2 (REVERBβ). The clock regulates the expression of both of these nuclear receptors, whereas their encoded proteins act to inhibit *BMAL1* transcription, thus providing temporal control of the core loop, as well as regulating the circadian expression of thousands of other genes, many associated with metabolism. The NR1Ds/REVERBs also drive the circadian recruitment of the deacetylase HDAC3, thus regulating the rhythmic modification of chromatin and consequent repression of transcription at REVERB binding sites 11, 12. The clock has many other molecular links with cellular metabolism and responds to the metabolic state of the cell. For example, NAD+/NADH redox equilibrium is detected via direct binding of these metabolites to CLOCK or NPAS2 and thereby modulating DNA binding of the CLOCK (NPAS2)/BMAL1 heterodimer 13. Cellular redox state can also be sensed by the REVERBs via heme binding, which enhances their interaction with the co‐repressors NCOR1 and HDAC3, thus regulating circadian transcription 14.

Using our own algorithm, we have previously analyzed transcriptomic data from human blood cells to identify genes that have a prevalent circadian expression profile in four separate conditions 2, 7. Thus, we assess each individuals’ time‐series separately to identify transcripts that are frequently observed, with a false discovery rate of less than 5%, to have a significant oscillatory component. Clearly, if we analyze and/or visualize the averaged (across subjects) transcriptomic profiles, where there is a dependency on expression value congruency between subjects, and/or change our selection thresholds, different results may be produced/observed.

### When sleep and the body clock are synchronized, 6.4% of genes in blood are rhythmic

**Figure 1:**
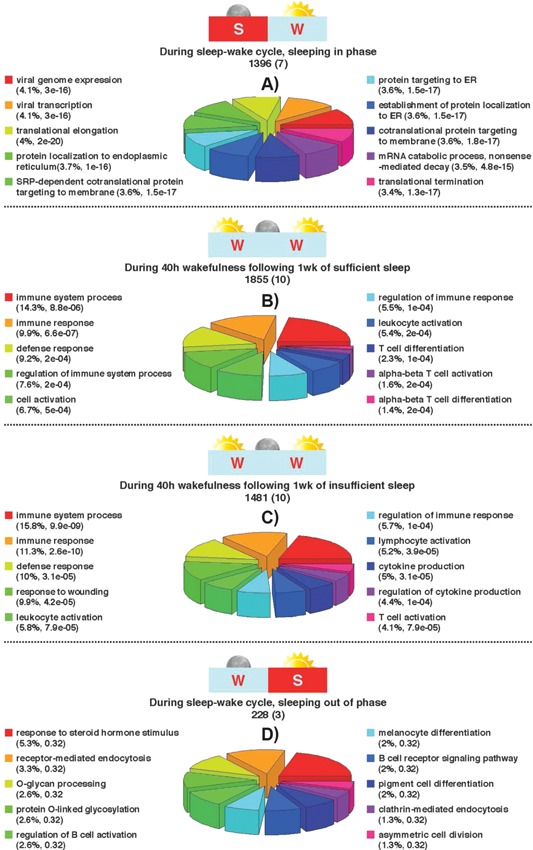
Number of genes with a circadian expression profile identified for each of the four sleep conditions; **A:** sleeping in phase with melatonin; **B:** during acute sleep deprivation after sufficient sleep; **C:** during acute sleep deprivation after insufficient sleep; **D:** sleeping out of phase with melatonin). Cartoons indicate when sleep (S) and wake (W) occurred with respect to biological day (sun) and biological night (moon). For each condition, numbers in brackets indicate the number of rhythmic core clock genes. The top‐ten biological processes associated with the genes with a circadian expression profile identified in each comparison listed above, respectively. The % enrichment for each process is listed together with the *p*‐value. The colour‐coded pie chart segments also represent the % enrichment for each process.

Under baseline conditions of sleeping in phase with the melatonin rhythm, 1,396 genes were characterized as having a circadian expression profile (Fig. 1A, Supplemental Table 1). These included the majority of the core circadian clock genes (Table 1). Gene ontology (GO) analysis using the online tool WebGestalt 15, 16 with default parameters and the human genome as background, showed that these genes with a circadian expression profile were associated with biological processes that mainly relate to the regulation of transcription and translation (Fig. 1A).

**Table 1:** Circadian rhythmicity of core clock genes across human and mouse samples

								Human samples							Mouse samples															
Human gene symbol	Mouse gene symbol	Total human lists	Total mouse lists	Percent of all human samples %	Percent of human blood samples %	Percent of all mouse samples %	Percent of all samples %	A	B	C	D	E	F	G	H	I	J	K	L	M	N	O	P	Q	R	S	T	U	V	W
NR1D1	Nr1d1	6	16	86	75	100	96	1	0	1	1	1	1	1	1	1	1	1	1	1	1	1	1	1	1	1	1	1	1	1
NR1D2	Nr1d2	6	16	86	100	100	96	1	1	1	1	0	1	1	1	1	1	1	1	1	1	1	1	1	1	1	1	1	1	1
PER3	Per3	6	15	86	75	94	91	1	1	0	1	1	1	1	1	0	1	1	1	1	1	1	1	1	1	1	1	1	1	1
ARNTL	Arntl	5	16	71	75	100	91	0	1	0	1	1	1	1	1	1	1	1	1	1	1	1	1	1	1	1	1	1	1	1
PER2	Per2	5	16	71	75	100	91	0	1	0	1	1	1	1	1	1	1	1	1	1	1	1	1	1	1	1	1	1	1	1
PER1	Per1	5	15	71	50	94	87	1	0	0	1	1	1	1	1	0	1	1	1	1	1	1	1	1	1	1	1	1	1	1
NPAS2	Npas2	3	15	43	75	94	78	0	1	0	0	0	1	1	1	1	1	1	1	1	1	1	1	1	0	1	1	1	1	1
CRY1	Cry1	2	13	29	0	81	65	0	0	0	1	1	0	0	1	1	1	1	1	1	0	1	0	1	0	1	1	1	1	1
CLOCK	Clock	1	13	14	25	81	61	0	0	0	0	0	0	1	1	1	1	0	1	1	0	1	1	1	0	1	1	1	1	1
CRY2	Cry2	2	10	29	25	63	52	0	1	0	1	0	0	0	1	0	1	0	1	1	0	1	1	1	0	1	0	1	1	0
CSNK1E	Csnk1e	5	2	71	100	13	30	0	1	1	1	0	1	1	0	0	0	0	0	0	0	0	0	1	0	0	0	1	0	0
RORA	Rora	2	2	29	50	13	17	0	0	0	0	0	1	1	1	0	0	0	0	0	0	0	0	0	0	0	0	1	0	0
CSNK1D	Csnk1d	1	2	14	25	13	13	0	0	0	0	0	1	0	0	1	0	0	0	0	0	0	0	0	0	0	1	0	0	0

Key for samples, 1 indicates rhythmic; 0 indicates arrhythmic; A, Akashi follicle (240 genes); B, Archer D1 (1395 genes); C, Archer D2 (228 genes); D, Hughes (1111 genes); E, Li Human brain (916 genes); F, Moller‐Levet SE (1855 genes); G, Moller‐Levet SR (1480 genes); H, Barclay rhythmic control classes123 (3231 genes); I, Barclay rhythmic TSR classes126 (2461 genes); J, Maret Table3 Control Condition (1654 genes); K, Maret Table4 Sleep Deprivation (340 genes); L, Hogenesch Adrenal gland (1052 genes); M, Hogenesch Aorta (873 genes); N, Hogenesch BrainStem (693 genes); O, Hogenesch Brown fat (1492 genes); P, Hogenesch Cerebellum (726 genes); Q, Hogenesch Heart (1187 genes); R, Hogenesch Hypothalamus (642 genes); S, Hogenesch Kidney (2603 genes); T, Hogenesch Liver (3186 genes); U, Hogenesch Lung (2327 genes); V, Hogenesch Skeletal Muscle (733 genes); W, Hogenesch white fat (856 genes).

### One week of insufficient sleep reduces the number of rhythmic transcripts by ∼20%

During 40 hours of TSD after either 1 week of sufficient sleep (ca. 8.5 hours per night) or after 1 week of insufficient sleep (ca. 5.7 hours per night), there were similar numbers [1,855 (8.8%) and 1,481 (6.9%), respectively] of genes with a circadian expression profile (Fig. 1B and C). In both of these conditions, the majority of the core circadian clock genes were also rhythmic (Table 1). However, unlike when sleeping in phase with melatonin (i.e. in phase with the central hypothalamic clock), genes that were rhythmic in both of these conditions of TSD were associated with blood‐related, day‐active biological processes that included immune response, defence response, leukocyte activation, and T cell activation.

### Only 1% of blood genes are rhythmic when sleep occurs out of phase with the body clock

When sleep occurred out of phase with melatonin, only 228 (1%) genes remained rhythmic, including only three core clock genes (*NR1D1*, *NR1D2*, and *CSK1ε*) (Fig. 1D). With this reduced number of rhythmic genes, none of the GO terms identified were significant but the top‐ten were related to some blood‐specific functions (Fig. 1D).

### 826 rhythmic genes are invulnerable to one week of sleep loss

**Figure 2:**
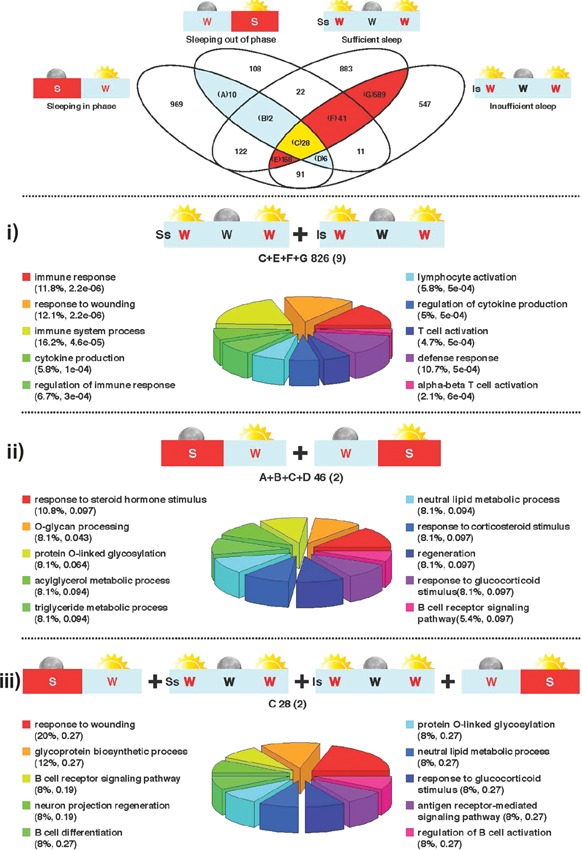
Venn diagram depicting the overlap of the lists of genes with a circadian expression profile obtained from analysis of gene expression profiles in each of the four sleep conditions tested using our previously reported algorithm 2, 7. Orange plus yellow highlighted area indicates genes that are identified as having a circadian expression profile during 40 hours of wakefulness with and without sufficient sleep. Blue and yellow highlighted area indicates genes that are identified as having a circadian expression profile when sleeping in and out of phase with melatonin. Yellow area indicates genes that are found to have a circadian expression profile across all conditions we have tested in humans, i–iii. Top‐ten biological processes associated with the circadian genes identified in each comparison listed above, respectively. Letters indicate which segments of the Venn contributed to the comparison. The total number of circadian genes identified is given together with the number of rhythmic core clock genes (in brackets). The % enrichment for each process is listed together with the *p*‐value. The colour‐coded pie chart segments also represent the % enrichment for each process.

**Figure 3:**
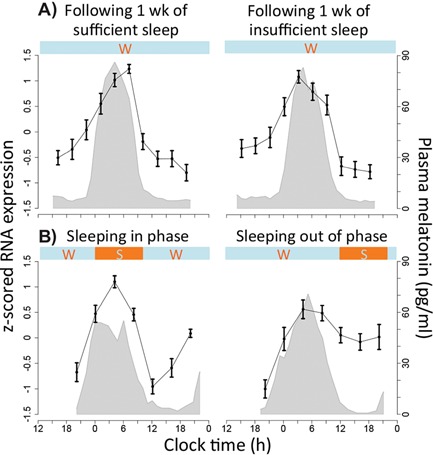
The z‐scored mRNA expression level (mean ± SEM across subjects) of *NR1D2* (A_23_P302709) within human blood during our investigations of **A:** chronic and acute sleep loss, **B:** forced desynchrony. Shaded grey area indicates the corresponding average (across subjects) plasma melatonin profile. Horizontal bars indicate wake (w) and sleep (s) periods. The figure shows that the expression profile of *NR1D2* remains relatively unchanged in response to both insufficient sleep (**A**) and mistimed sleep (**B**) with a peak of expression close to the peak of melatonin secretion.

**Figure 4:**
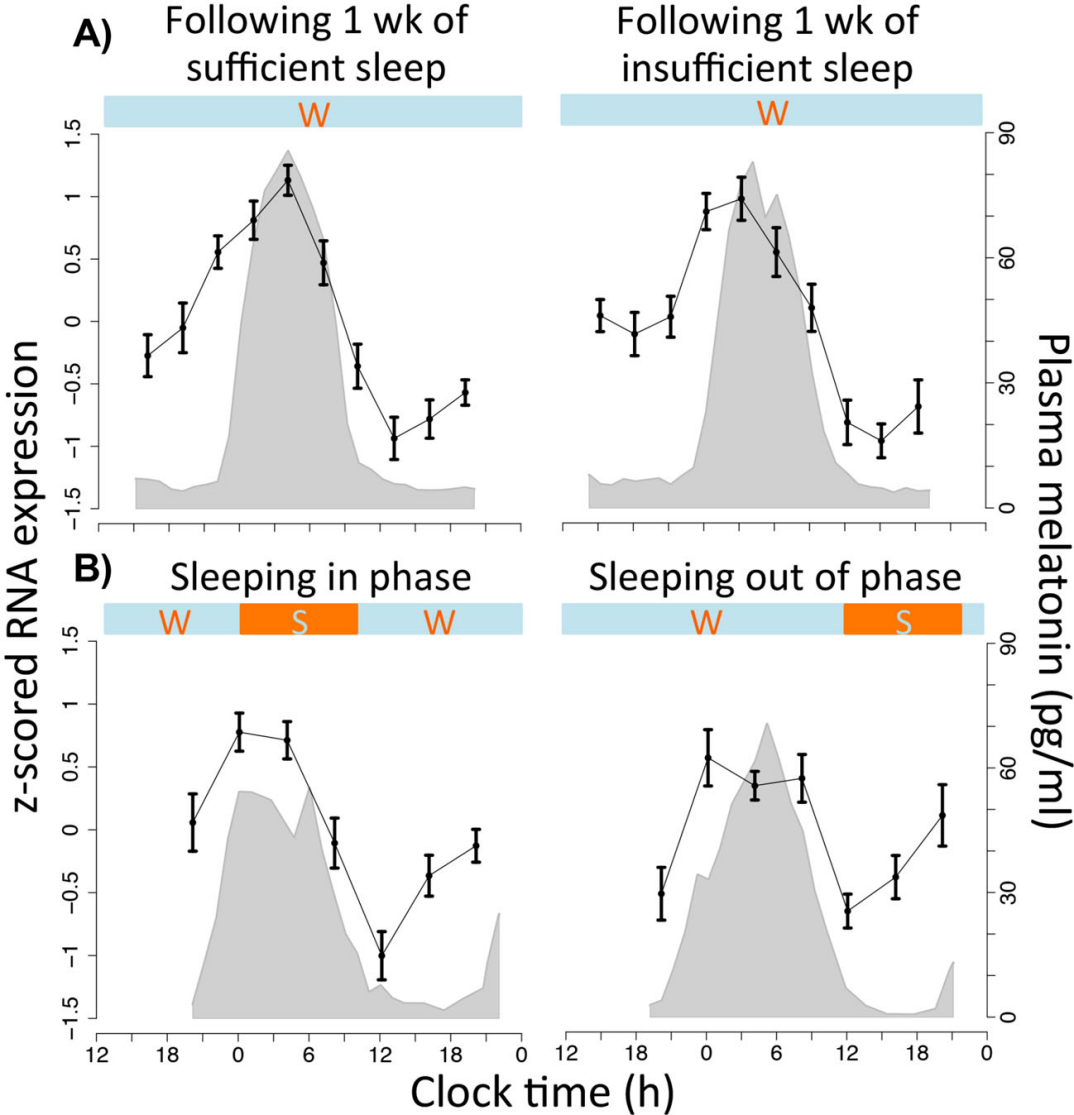
The z‐scored mRNA expression level (mean ± SEM across subjects) of *NR1D1* (A_23_P420873) within human blood during our investigations of **A:** chronic and acute sleep loss, **B:** forced desynchrony. Shaded grey area indicates the corresponding average (across subjects) plasma melatonin profile. Horizontal bars indicate wake (w) and sleep (s) periods. The figure shows that the expression profile of *NR1D1* remains relatively unchanged in response to both insufficient sleep (**A**) and mistimed sleep (**B**) with a peak of expression close to the peak of melatonin secretion.

Following the description of genes with rhythmic expression in each of the four experimental conditions (Fig. 1), we next assessed rhythmic genes common to different conditions (Fig. 2). In total, 826 genes (Supplementary Table 1) were found to have a circadian rhythm during the 40 hours TSD, irrespective of whether subjects had experienced one week of sufficient or insufficient sleep (Fig. 2i). Here the majority of the core circadian clock genes were rhythmic, including: *NR1D2* (Fig. 3), *NR1D1* (Fig. 4), *CSNK1*ε, *ARNTL*, *NPAS2*, *PER1*, *PER2*, *PER3*, and *RORA*; *CSNK1δ* was only rhythmic after 1 week of sufficient sleep, and CLOCK was only rhythmic after 1 week of insufficient sleep (Table 1). The top‐ten significant biological processes that are over‐represented in this gene list include: response to wounding, immune response, defense response, cytokine production, lymphocyte activation, and T cell activation (Fig. 2i).

### 46 rhythmic genes are invulnerable to desynchronized sleep

We then compared the conditions of sleeping in and out of phase with melatonin, a perturbation with substantial effects on rhythmicity within the human transcriptome 2. We found only 46 genes had a circadian expression profile in both conditions (Fig. 2ii, Supplementary Table 1). When sleeping in phase with melatonin, transcripts with a rhythmic expression profile included the core clock genes *NR1D2*, *CSNK1*ε, *ARNTL*, *NPAS2*, *PER2*, *PER3*, and *CRY2*, whereas when sleeping out of phase only *NR1D2* (Fig. 3) and *CSNK1*ε remained rhythmic while *NR1D1* became rhythmic (Fig. 4) (Table 1). Although *NR1D1* by our analytical approach is not statistically characterized as circadian when sleeping in phase, its expression profile nevertheless demonstrates similarity to other temporal profiles (Fig. 4). The top‐ten biological processes that are over‐represented in this gene list included: response to steroid hormone stimulus, O‐glycan processing, triglyceride metabolic process, neutral lipid metabolic process, response to corticosteroid stimulus, and the B cell receptor signalling pathway (Fig. 2ii).

### A core set of 28 genes remains rhythmic in blood during all four sleep conditions

Despite significant physiological differences between the four sleep conditions analyzed, in which the presence/absence of sleep and also the timing of sleep relative to the central circadian clock are investigated, 28 genes were identified as being rhythmic across all conditions (Fig. 2iii; Table 2; Supplementary Table 2). These “robust” circadian genes are rhythmically expressed independently from the sleep/wake cycle and must, at least in humans, constitute a central component of circadian processes in blood. This list includes only two canonical clock genes, namely *NR1D2* and *CSNK1*ε. The top‐ten, but not statistically significant, biological processes over‐represented in this robust circadian gene list include: response to wounding, neuron projection regeneration, the B cell receptor signalling pathway, response to glucocorticoid stimulus, sulfur compound biosynthetic process, immune response‐regulating cell surface receptor signalling pathway, O‐glycan processing, humoral immune response, neutral lipid metabolic process, and response to temperature stimulus.

**Table 2:** Human genes characterized as robustly rhythmic in human blood and their rhythmicity in other samples

								Human samples							Mouse samples															
Human gene symbol	Mouse gene symbol	Total human lists	Total mouse lists	Percent of all human samples %	Percent of human Blood samples %	Percent of all mouse samples %	Percent of all samples %	A	B	C	D	E	F	G	H	I	J	K	L	M	N	O	P	Q	R	S	T	U	V	W
NR1D2	Nr1d2	6	16	86	100	100	96	1	1	1	1	0	1	1	1	1	1	1	1	1	1	1	1	1	1	1	1	1	1	1
SLC6A6	Slc6a6	5	8	71	100	50	57	0	1	1	1	0	1	1	0	1	0	0	1	1	1	1	0	1	0	0	0	1	0	1
FAM126B	Fam126b	4	6	57	100	38	43	0	1	1	0	0	1	1	1	0	0	0	0	0	1	0	0	0	1	0	1	1	1	0
MPZL1	Mpzl1	4	6	57	100	38	43	0	1	1	0	0	1	1	0	0	0	0	1	0	0	0	0	1	1	1	1	1	0	0
HNRPDL	Hnrpdl	4	5	57	100	31	39	0	1	1	0	0	1	1	0	0	1	0	0	0	1	0	0	1	0	1	1	0	0	0
TMEM140	Tmem140	4	5	57	100	31	39	0	1	1	0	0	1	1	1	0	0	0	1	0	0	1	0	0	0	0	0	1	1	0
B4GALT5	B4galt5	6	2	86	100	13	35	0	1	1	1	1	1	1	0	0	0	0	0	0	0	0	0	0	0	0	1	1	0	0
GCA	Gca	4	4	57	100	25	35	0	1	1	0	0	1	1	0	1	0	0	0	0	0	1	0	0	0	0	1	1	0	0
CSNK1E	Csnk1e	5	2	71	100	13	30	0	1	1	1	0	1	1	0	0	0	0	0	0	0	0	0	1	0	0	0	1	0	0
CCNJL	Ccnjl	4	3	57	100	19	30	0	1	1	0	0	1	1	0	0	1	0	0	0	0	0	0	1	0	0	0	1	0	0
ST6GALNAC2	St6galnac2	4	3	57	100	19	30	0	1	1	0	0	1	1	0	0	0	0	1	0	0	1	0	0	0	0	0	1	0	0
ADM	Adm	4	2	57	100	13	26	0	1	1	0	0	1	1	0	0	0	0	0	0	0	0	0	1	0	0	0	1	0	0
BCL2	Bcl2	4	2	57	100	13	26	0	1	1	0	0	1	1	0	0	1	0	0	0	0	0	0	0	0	0	0	0	1	0
SLC22A4	Slc22a4	4	2	57	100	13	26	0	1	1	0	0	1	1	0	0	0	0	0	1	0	0	0	0	0	1	0	0	0	0
GK	Gyk	4	1	57	100	6	22	0	1	1	0	0	1	1	0	1	0	0	0	0	0	0	0	0	0	0	0	0	0	0
MAL	Mal	4	1	57	100	6	22	0	1	1	0	0	1	1	0	0	0	0	0	0	0	0	0	0	0	0	0	1	0	0
ALOX5AP	Alox5ap	4	0	57	100	0	17	0	1	1	0	0	1	1	0	0	0	0	0	0	0	0	0	0	0	0	0	0	0	0
AVIL	Avil	4	0	57	100	0	17	0	1	1	0	0	1	1	0	0	0	0	0	0	0	0	0	0	0	0	0	0	0	0
CNTNAP3	Cntnap3	4	0	57	100	0	17	0	1	1	0	0	1	1	0	0	0	0	0	0	0	0	0	0	0	0	0	0	0	0
DAAM2	Daam2	4	0	57	100	0	17	0	1	1	0	0	1	1	0	0	0	0	0	0	0	0	0	0	0	0	0	0	0	0
NELL2	Nell2	4	0	57	100	0	17	0	1	1	0	0	1	1	0	0	0	0	0	0	0	0	0	0	0	0	0	0	0	0
NFAM1	Nfam1	4	0	57	100	0	17	0	1	1	0	0	1	1	0	0	0	0	0	0	0	0	0	0	0	0	0	0	0	0
OTX1	Otx1	4	0	57	100	0	17	0	1	1	0	0	1	1	0	0	0	0	0	0	0	0	0	0	0	0	0	0	0	0
TREM1	Trem1	4	0	57	100	0	17	0	1	1	0	0	1	1	0	0	0	0	0	0	0	0	0	0	0	0	0	0	0	0
A_24_P725998	NA	4	NA	57	100	NA	17	0	1	1	0	0	1	1	0	0	0	0	0	0	0	0	0	0	0	0	0	0	0	0
A_32_P133564	NA	4	NA	57	100	NA	17	0	1	1	0	0	1	1	0	0	0	0	0	0	0	0	0	0	0	0	0	0	0	0
HCG27	NA	4	NA	57	100	NA	17	0	1	1	0	0	1	1	0	0	0	0	0	0	0	0	0	0	0	0	0	0	0	0
CREB5	Creb5	4	NA	57	100	NA	17	0	1	1	0	0	1	1	0	0	0	0	0	0	0	0	0	0	0	0	0	0	0	0

Key for samples: A, Akashi follicle (240 genes), B, Archer Sleeping in Phase (1,395 genes); C, Archer Sleeping out of Phase (228 genes); D, Hughes (1,111 genes); E, Li Human brain (916 genes); F, Moller‐Levet Sleep Extension (1,855 genes); G, Moller‐Levet Sleep Restriction (1,480 genes); H, Barclay rhythmic control classes 1, 2, and 3 (3,231 genes); I, Barclay rhythmic Total Sleep Restriction classes 1, 2, and 6 (2,461 genes); J, Maret Table3 Control Condition (1,654 genes); K, Maret Table 4 Sleep Deprivation (340 genes); L, Hogenesch Adrenal gland (1,052 genes); M, Hogenesch Aorta (873 genes); N, Hogenesch BrainStem (693 genes); O, Hogenesch Brown fat (1,492 genes); P, Hogenesch Cerebellum (726 genes); Q, Hogenesch Heart (1,187 genes); R, Hogenesch Hypothalamus (642 genes); S, Hogenesch Kidney (2,603 genes); T, Hogenesch Liver (3,186 genes); U, Hogenesch Lung (2,327 genes); V, Hogenesch Skeletal Muscle (733 genes); W, Hogenesch white fat (856 genes).

### The majority of the 28 robustly rhythmic genes have peak expression during the biological day

Of the 28 genes we identify as rhythmic in all four human sleep conditions, 23 had peak expression during the biological day, i.e. when melatonin concentrations are very low, and only five (*NR1D2*, *HNRPDL*, *BCL2*, *MAL*, and *NELL2*) had expression peaks during the biological night. In agreement with our previous data, the day‐peaking robust genes were associated with processes related to immune function and included response to wounding, B cell receptor signalling, and humoral immune response. Thus, the core set of robust circadian genes are overwhelmingly expressed during the day and involved with blood‐specific immune responses. Of the five genes whose expression peak during the night, two are involved with binding to nucleic acid and regulating either transcription (*NR1D2*) or translation (*HNRPDL*). HNRPDL (heterogeneous nuclear ribonucleoprotein D‐like) binds NRF (NFκβ repressing factor) mRNA either at the 5′‐untranslated region (5′‐UTR) leading to increased translation initiation, or at its 3′‐UTR where it leads to increased mRNA stability 17. BCL2 is an anti‐apoptotic factor that forms part of the unfolded protein response within the endoplasmic reticulum stress rheostat pathway by regulating calcium flux and mitochondrial control of apoptosis 18. MAL (myelin and lymphocyte protein; T cell differentiation protein) forms part of the protein machinery that transports membrane and secretory proteins and is involved in membrane trafficking and signalling in T cells 19. NELL2 (neural epidermal growth factor‐like 2) plays a role in neuron differentiation but is also expressed in nucleated blood cells where it may function to regulate cell development 20. In the brain, NELL2 has been shown to regulate long‐term potentiation (LTP) and hippocampus‐dependent spatial learning in mice 21. Therefore, this small group of highly robust circadian night‐peaking genes are involved with the regulation of gene expression (in agreement with our previous data), the trafficking of newly synthesized proteins and responses to incorrect protein folding, and blood cell development. Together, these biological day‐ and night‐peaking genes represent core processes whose rhythmicity at the mRNA level remains insensitive to sleep loss and sleep displacement.

## Regulatory network driving expression of robust circadian genes

Given the independence of our 28 robust circadian genes from the sleep/wake cycle, it is of interest to investigate signals that could drive or at least influence their rhythmic expression. Based on analysis of current published data, using MetaCore^TM^ (Thomson Reuters) and/or STRING (http://string‐db.org/), no direct interactions between the members of our group of 28 human circadian genes and their products are known. We therefore adopted two approaches to investigate the putative regulatory networks that could be created by looking at molecular interactions such as protein‐DNA (e.g. transcription factor binding) and/or protein‐protein interactions, directly upstream of the 28 genes, i.e. interactions where one of the 28 genes (or their products) are considered to be the target. To identify interactions, we used the following criteria. The first approach employed the “build network” tool in MetaCore. In the second approach we interrogated the ENCODE Uniform ChIP‐seq dataset via the University of California Santa Cruz (UCSC) genome browser (data last modified on April 12 2013). Five hundred and eighty‐four data files containing the genomic coordinates of binding peaks for 161 transcription factors within different untreated human cell lines were obtained. The peak coordinates were compared with the 1,000 bp upstream regions of all genes (also obtained from UCSC), and those genes with an upstream region containing a transcription factor‐binding peak were classed as targets of that transcription factor.

Twenty‐three of the 28 robust circadian genes had upstream interactions documented within MetaCore (accessed May 11 2014). The network comprising these genes and their upstream “interactors” consists of 14,649 interactions (note that this number includes interactions between upstream interactors). Focusing on the 516 interactions where the target (as opposed to source) of an interaction was one of the robust circadian genes, we discovered that no single upstream element interacted with more than 6 of the 28 robust circadian genes, suggesting that there are likely to be multiple “core” output pathways closely linked to the clock and relatively insensitive to sleep. However, we did find nine upstream elements that interacted with at least four (17%) of these genes: estrogen receptor 1 (ESR1; six genes, 26%), UBIQUITIN (six genes, 26%), cyclic AMP response element binding protein 1 (CREB1; five genes, 22%), TIF1B (five genes, 22%), OCT3/4 (four genes, 17%), CJUN (four genes, 17%), SP1 (four genes, 17%), CMYC (four genes, 17%), and GCRA (four genes, 17%).

### Six of the core rhythmic genes interact with estrogen receptor 1

Two of these nine upstream elements are worthy of more detailed discussion, due to their known links with circadian physiology. Six of the core rhythmic genes (*BCL2*, *NELL2*, *SLC6A6*, *DAAM2*, *OCTN1*, *ADM*) have direct interactions with ESR1. ESR1 has been shown to regulate sex‐specific circadian behavior in mice. Female mice have greater levels of activity than males but this difference is absent in *Esr1* knock out animals 22. Female *Esr1* knock‐out mice also have a smaller phase shift in response to a light pulse in the subjective night and this difference is absent in *Esr1* knockout mice 23. In our forced desynchrony protocol, *ESR1* was down regulated and was the most connected node in a direct interaction network of genes that showed a main effect of sleep condition 2. *ESR1* was also the most connected node in an interaction network constructed from genes that showed differential promoter methylation in shift workers 24. Thus, ESR1 shows consistent links with pathways that are associated with, and are affected by manipulations of sleep‐wake activity.

### Multiple factors interact to determine circadian expression

It may seem counter intuitive that *ESR1* is down regulated during mistimed sleep but shows interaction with six of the core circadian genes. We can only speculate on how ESR1 influences the expression of these genes. *ESR1* expression is down regulated with mistimed sleep but the transcriptional repressors *NCOR1* and *REST* that regulate ESR1 mediated gene expression are more down regulated during mistimed sleep and specifically during the day 2. Thus, overall levels of gene expression driven by ESR1 could be higher. However, several factors can interact to determine the overall expression profiles of circadian genes. We have previously modeled the interaction between positive or negative drives from melatonin and sleep, which can then predict the expression profiles of transcripts when sleep occurs in or out of phase with melatonin 2. For example, if the expression of a circadian gene is suppressed by melatonin (peaks during the day) but enhanced by sleep, the expression profile can be circadian when sleep occurs in phase with melatonin but with reduced amplitude compared to when sleep occurs out of phase with melatonin. Indeed, several of the core circadian genes in our previous analysis 2 fell into this category (e.g. *SLC6A6* and *CSNK1E*) and the majority of the core circadian genes in this analysis do too.

### Five of the core rhythmic genes interact with the circadian entrainment mediator CREB

Of relevance to other known clock‐related pathways, five genes (*CREB5*, *BCL2*, *HNRNPDL*, *GK*, and *DAAM2*) have direct interactions with the transcription factor CREB1. Light‐induced phosphorylation of CREB in the mouse SCN allows it to bind to cAMP response element sites in the promoters of *Per1* and *Per2*, thus providing an entrainment signal for the central circadian clock 25. CREB is also an activity‐dependent transcription factor in the brain where levels in the cortex are higher during wake than sleep and contribute towards sustaining wakefulness 26. In the periphery, CREB regulates glucose homeostasis and gluconeogenesis in the liver 27, and has multiple roles in the immune system, including regulation of genes such as *IL2*, *IL6*, *IL10*, and *TNFα*, inhibition of NFκβ, regulating the proliferation and survival of T and B cells, and the generation of regulatory T cells 28. When CREB is activated by phosphorylation, it interacts with its coactivator proteins CREBBP (CREB binding protein) and EP300, which stimulate transcription by modifying DNA through histone acetylation. Rhythmic recruitment of CREBBP and EP300 has been shown to play an integral part in the circadian regulation of the mammalian transcriptome 29. Related to this, it is perhaps particularly relevant that one of the 28 robust circadian genes is CREB5.

Our analyzes indicate that multiple upstream regulatory pathways may drive the rhythmic expression of the most robust circadian genes. Some of these pathways have been reported by others, but it is likely that integrated circadian gene expression requires the coordinated input of multiple pathways, some of which may not have been previously linked to circadian biology. Alternatively, it is possible that the power of this analytical approach and the datasets used do not allow the identification of more universal upstream regulatory transcription factors. Lastly, circadian rhythmicity in gene expression is highly tissue‐specific: many genes (∼43%) are rhythmic in at least one tissue 9 but perhaps only certain elements of the core clock are robustly rhythmic across many tissues.

## Congruency of human and mouse rhythmic gene expression signals

To identify circadian genes across different data sets that are publicly available from the CIRCA database 8, 9, we used the selection criteria of: at least one probe with a JTK q‐value and/or *p*‐value < 0.05 4. Using this threshold, we identified 1,111 unique genes identified as having a circadian expression profile in the human bone tissue cell line U2OS. By examining the recently added RNA‐seq data sets comprising global transcriptome measurements across 12 different mouse tissues 1, we then identified 13,644 unique mouse genes that had a circadian expression profile in at least one tissue stored within the CIRCA database. We also analyzed the circadian gene lists from human hair follicles (240 genes) 1, post‐mortem human brain tissue (916 genes in the dorsolateral prefrontal cortex) 5, and from two mouse sleep deprivation studies in brain (1,654 genes in control sleep condition and 340 genes in sleep deprivation condition) 6 and liver (3,231 genes for control, and 2,461 genes for sleep restriction) 3.

### Most canonical clock genes are rhythmic across most samples

Ten of the 13 canonical clock genes listed in Table 1 are classified as having rhythmic expression profiles in at least 50% of all samples from human and mouse. Several of these (*NR1D1, NR1D2, PER2, PER3*, and *ARNTL*) are rhythmic in over 90% of all samples analyzed and thus represent the robust core of the circadian molecular clockwork. If rhythmic genes are sorted according to the percentage of all samples in which genes are rhythmic (Supplemental Table 1), there are 32 genes that are rhythmic in at least 52% of all human and mouse samples. Within these 32, there are 10 canonical clock genes, two clock output transcription factors (*DBP* and *TEF*), and the circadian transcriptional repressor, *BHLHE41* (*DEC2*). Some clock genes appear to show species‐specific rhythmicity; *CRY1,* *CRY2*, and *CLOCK* are rhythmic in few human samples but highly rhythmic in the mouse samples, whereas *CSNK1* *ε* is rhythmic in 71% of all human samples but only 13% of all mouse samples. By far the largest effect of sleep disruption on clock gene expression rhythms is seen when sleep was displaced by 12 hours from the biological clock, where only three clock genes remained rhythmic (*NR1D1*, *NR1D2*, and *CSNK1* *ε*) 2.

### The core set of 28 robust human circadian genes invulnerable to sleep disruption is blood‐specific

Of the 28 human genes that remain rhythmic in blood across the four different sleep conditions, only two (*NR1D2* and *SLC6A6*) are also rhythmic in at least 50% of the mouse samples, and the majority are also not rhythmic in the human hair, bone, and brain samples (Table 2). As mentioned, these blood‐specific genes are enriched with transcripts that are associated with blood‐specific immune functions. Because genome‐wide expression has not been measured in mouse blood, future investigations will need to determine if these also represent human‐specific blood transcripts. Similarly, the limited accessibility of human tissues could influence data interpretation.

### A subset of genes is circadian across the majority of human samples

Four genes (*NR1D2*, *B4GALT5*, *SLC6A6*, and *CSNK1*ε) were circadian across all human sleep conditions and in at least one other human data set. B4GALT5 (β‐1, 4‐galactosyltransferase) transfers sugar moieties in the biosynthesis of glycans and regulates the hedgehog signalling pathway that controls cell development and proliferation 30. SLC6A6 (solute carrier family six member 6–neurotransmitter transporter; also called TAUT) is a taurine transporter. Taurine does not become incorporated into proteins but regulates many cellular functions including cell volume, antioxidant defense, protein stabilization, stress response, and immunomodulation (see 31). CSNK1ε phosphorylates PER proteins and plays an important role in the circadian clock. Its temperature‐insensitive activity can determine circadian period 10 and its absence in CSNK1δ‐deficient cells abolishes circadian rhythmicity 32. However, CSNK1ε is a key phosphorylating enzyme that provides post‐translational regulation of many other important pathways. These include the WNT signaling pathway important during development and cancer 33, 34, the modulation of topoisomerase and DNA cleavage activity 35, and the regulation of the binding activity of DNA methyltransferase I 36. The fact that CSNK1ε functions in a temperature‐insensitive way may be relevant to why it, and possibly other robust circadian genes, remains robustly rhythmic during all four sleep conditions, including during mistimed sleep, when the amplitude of core body temperature is known to be reduced 37.

### *NR1D1* and *NR1D2* are the two most rhythmic genes across all human and mouse samples

Mapping gene names through the tool MadGene 38, we found that 15 (65%, not including genes with no apparent homolog) of the 28 human robust circadian genes had identifiable homologs in mouse that were circadian in at least one tissue. Of the four genes that were circadian in at least 71% of all human tissues and sleep conditions, only two were also highly rhythmic across mouse tissues and sleep conditions [*NR1D2* (100%) and *SLC6A6* (50%)] (Table 2). Only one gene, *NR1D2,* was found to be circadian across the majority of human and mouse tissues (96%) and in all sleep conditions (control, sleep restriction, and mistimed sleep in both humans and mice) (Table 2, Fig. 3 and Supplementary Figure 1). The related gene *NR1D1* exhibited similarly robust circadian rhythms, although without reaching statistical significance in one of the human blood data sets. Nevertheless, when genes are ranked according to the percentage of all samples where they are rhythmic (Supplemental Table 1), *NR1D1* and *NR1D2* are the highest ranking genes both being rhythmic in ∼96% of all human and mouse samples.

The functional roles of *NR1D1* and *NR1D2* within the circadian clock have recently been identified. A role for *NR1D1* within the mammalian clock machinery was proposed over 10 years ago 39. More recent work with *Nr1d1/Nr1d2* null mice 12 and RNA silencing 40 now point to *NR1D2* having a more prominent and possibly more important cell type‐dependent role than previously thought. NR1D2 binds to Reverb/Ror element (RRE) sites in promoter elements. Peak binding occurring in mouse towards the end of the inactive light phase, 10 hours after “lights‐on” when *NR1D2* gene expression is highest 12, 41. In our human data, peak *NR1D2* gene expression also occurred towards the end of the biological night. The majority of the *NR1D2* genomic binding sites overlap with those of *NR1D1* and many are also shared with the BMAL1 cistrome and promoter recruitment sites for the nuclear receptor corepressor (NCOR1) and the histone deacetylase (HDAC3) transcriptional repressor complex 12, 41. Thus, NR1D2/NR1D1 together with NCOR1/HDAC3 can suppress the transcription of *Bmal1* and genes whose expression is activated by BMAL1. Promoter regions that bind *NR1D2*/*NR1D1* are located in genes that are predominantly associated with primary metabolic processes, lipid metabolism, PPAR signalling, protein metabolic processes, and nucleic acid metabolic processes 12, 41.

The reason why *NR1D1* and *NR1D2* are at the top of ranked lists of rhythmic genes is unclear. We speculate that the ranking likely reflects the position of *NR1D1* and *NR1D2* and the interface of the circadian transcription‐translation loops and metabolic rhythms. Indeed, in liver‐specific knockdowns of both *Nr1d1* and *Nr1d2* in mice, more than 90% of genes whose expression is circadian lose rhythmicity, and animals display increased circulating glucose and triglyceride levels, and reduced levels of free‐fatty acid 41. Given the obvious importance of metabolism to cell and organism physiology, we therefore contend that *NR1D2*, possibly acting with *NR1D1*, provides robust timekeeping for key metabolic processes, and protects against state manipulations, such as changes in the occurrence and timing of sleep. Furthermore, other canonical circadian genes (e.g. *PER3*, *ARNTL*, *PER2*, *PER1*; Table 1) exhibit similar rhythmicity, albeit slightly less prominent than *NR1D1* and *NR1D2*. The independent finding by ourselves and others 9 that *NR1D1* and *NR1D2* are the most rhythmic genes indicates that the rhythmic function of the overall circadian clockwork is not only a feature of multiple tissues, but also remarkably resilient to sleep manipulations.

### Rhythmic genes affected by sleep disruption point towards mechanisms that underlie associated adverse health outcomes

**Figure 5:**
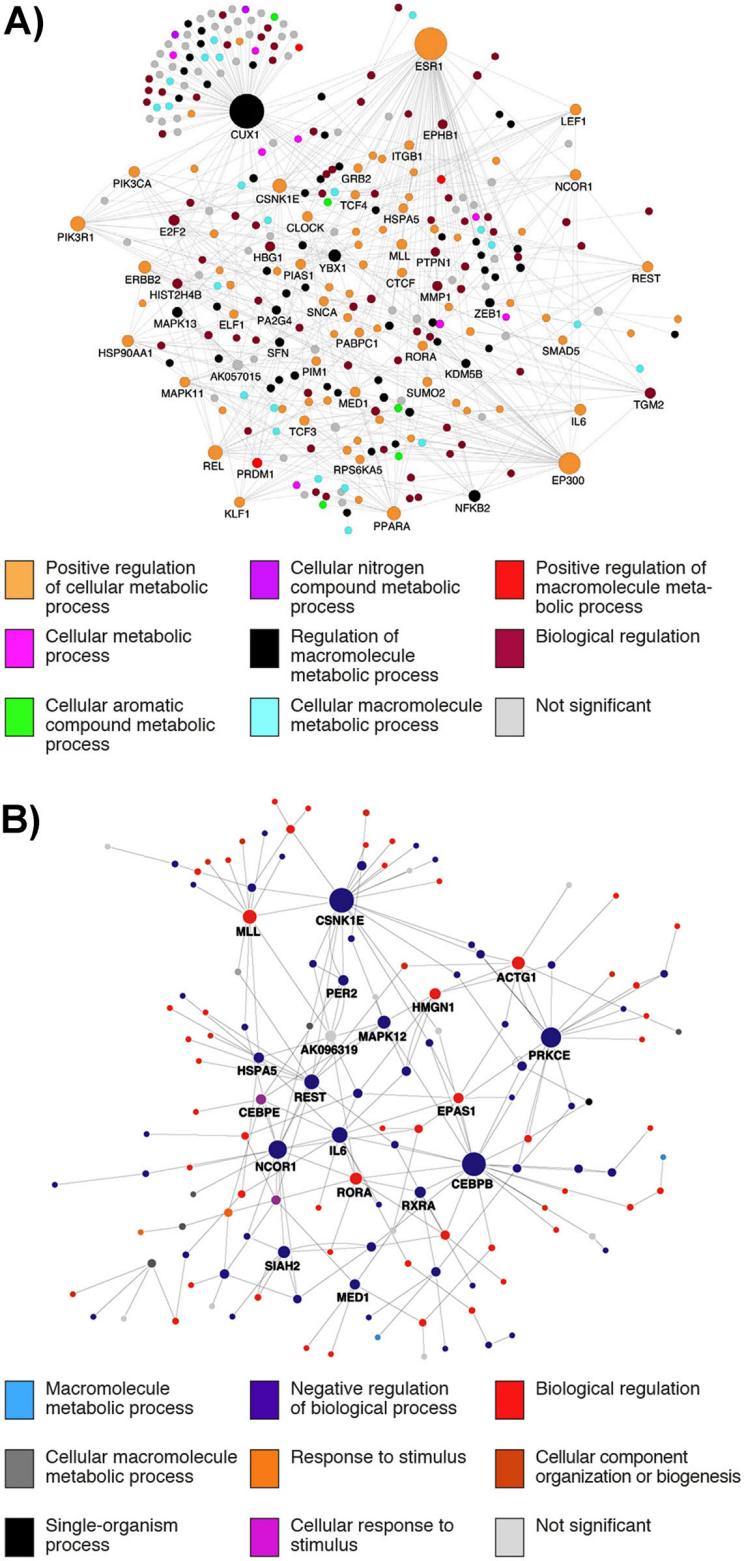
Direct interaction networks, as documented by MetaCore^TM^, between genes for which at least one transcript had a statistically significant main effect of sleeping in‐phase and out‐of‐phase with melatoni​n (**A**), or a main effect one week of either sufficient or insufficient sleep prior to a 40 hours period of total sleep deprivation (**B**). Node size reflects the number of direct connections a gene has within the network. Node color (see key) represents the most significantly enriched GO biological process term associated with that node given the submission of the statistically significant gene lists to the tool WebGestalt. The figures identify networks of genes that are affected by sleep disruption that also show high levels of interactivity. Some interacting genes are common to both sleep disruption conditions, and a large number are linked with processes known to be associated with adverse health outcomes (see text for details).

Analysis of variance for the expression profiles of genes in our two human‐blood sleep studies showed that there was a main effect of sleep condition in 744 transcripts when comparing sufficient and insufficient sleep 7 and for 1,119 transcripts comparing sleep in phase and sleep out of phase with melatonin 2. Using MetaCore^TM^ we performed a direct interaction network analysis on these two gene sets. This analysis connects elements that are known to interact at the gene or protein level. Major nodes within each interaction network are labelled, and node size indicates level of connectivity, while node colour indicates associated GO terms (Fig. 5). The interaction network for the ANOVA main effect of sleeping in out of phase with melatonin is shown in Fig. 5A, and for the main effect of sufficient or insufficient sleep in Fig. 5B. The greater disruption caused by sleeping out of phase with melatonin is reflected by a larger number of interacting elements (Fig. 5A). Many of these interaction nodes are associated with adverse health phenotypes: CUX1 repairs DNA oxidative damage and is a tumour suppressor 42, 43, REL and NFKB2 are part of the NFKB signalling pathway and associated with immune function and cancer 44, 45; REST is a transcriptional repressor linked with prostate cancer and regulates cardiac gene expression 46, 47; EP300 is a transcriptional co‐activator that regulates circadian gene expression and ESR1‐stimulated expression and is linked with prostate cancer 29, 48; IL6, and MAPK11 are linked with immune function and cancer 49, 50; PIK3CA and PIK3R1 are linked with insulin resistance, immune response and cancer 51, 52, 53, PPARA is associated with lipid homeostasis, inflammation, and type‐2 diabetes 54, 55, 56, MLL is a methyltransferase that regulates circadian gene expression and is linked with cancer 57, 58, and ESR1 regulates gene expression and is associated with cancer 59, 60. An overlapping set of interacting genes are affected by both sleep restriction and mistimed sleep: MLL, CSNK1ε, MAP kinase transcripts (MAPK11, MAPK12, MAPK13), HSPA5, REST, NCOR1, IL6, MED1, and RORA. Of interest, some of these genes and others in the interaction networks are intimately involved in the regulation of the RNA polymerase II transcriptional complex. MED1 forms a pivotal element of the RNA polymerase II Mediator complex 61. It interacts directly with thyroid, estrogen, and glucocorticoid receptor elements in gene promoters 61. The Mediator complex activity is modulated by MAP kinase 62 and DNA binding of the complex in promoter regions is modulated by NCOR1, EP300, and REST 61, 63. We have previously shown that sleeping out of phase with melatonin affects many biological processes and molecular functions associated with the regulation of transcription and translation 2. Here, the ANOVA main effect interaction networks from both of our sleep disruption studies show how specific elements that form an integrative regulatory hub for translation are affected. Many of these elements have links with cancer and MED1 has specific links with breast cancer 62. Therefore, we speculate that genes whose expression profiles become dysregulated during disrupted sleep, as occurs when sleep is displaced during shift work, point towards underlying biological processes that are linked with some of the adverse health outcomes associated with shift work, such as cancer and metabolic disorders.

## Conclusions and outlook

Resonance of robust, high‐amplitude circadian rhythms with behavioral cycles of sleep‐wake and fasting‐feeding are important for lifelong well‐being and health. Disruption to these synchronized rhythms, as occurs in shift work and ageing, leads to adverse health outcomes. Here, our results demonstrate that comparative (meta) analyses of multiple circadian transcriptomes provides novel insights into underlying circadian mechanisms. Furthermore, it will be useful to include data from additional human tissues/cells and other molecular technologies (e.g. proteomics, cistromics, and metabolomics). Such analyzes would also be expected to link disrupted circadian rhythms to negative health outcomes such as metabolic disorders (obesity, type 2 diabetes), cardiovascular disease, and cancer.

## Author contributions

D.J.D., E.E.L., J.D.J., and S.N.A. designed the research; E.E.L., C.M.L., S.N.A., G.B., C.P.S., and D.J.D., collected and analysed the data; E.E.L., J.D.J., D.J.D., and S.N.A., wrote the paper.

## Supporting information

As a service to our authors and readers, this journal provides supporting information supplied by the authors. Such materials are peer reviewed and may be re‐organized for online delivery, but are not copy‐edited or typeset. Technical support issues arising from supporting information (other than missing files) should be addressed to the authors.

**Table S1:** Circadian rhythmicity of genes in human and mouse samples.

**Table S2:** Annotations for the 28 genes identified as being robustly rhythmic in human blood samples.
