# Supplementary material for: Exploiting human and mouse transcriptomic data: Identification of circadian genes and pathways influencing health
Source: Bioessays. 2015 Mar 14;37(5):544–56. doi: 10.1002/bies.201400193 (PMC5031210; doi:10.1002/bies.201400193)
Supplement: Supplementary file 2 — Table S2: Annotations for the 28 genes identified as being robustly rhythmic in human blood samples. [file BIES-37-544-s002.doc]

**Supplementary table 2**, annotations for the 28 genes identified as being robustly rhythmic in human blood samples.

| **Gene names (Location)** | **Annotation (as taken from MetaCore)** |
| --- | --- |
| A_24_P725998 (GRCh37: Chromosome 9, 71150053..71150112, complement)  GRCh36: chr9:070339932-070339873 | TMEM252 |
| A_32_P133564 (GRCh37: Chromosome 17, 39077650-39077709, complement)  GRCh36: chr17:036331235-036331176 | None found |
| ADM (Chromosome 11, 10326642..10328923) | Adrenomedullin, a hypotensive peptide found in human pheochromocytoma, consists of 52 amino acids, has 1 intramolecular disulfide bond, and shows a slight homology with the calcitonin gene-related peptide. It may function as a hormone in circulation control because it is found in blood in a considerable concentration. |
| ALOX5AP (Chromosome 13, 31287615..31338565) | This gene encodes a protein which, with 5-lipoxygenase, is required for leukotriene synthesis. |
| AVIL (Chromosome 12, 58190415..58210193, complement) | The protein encoded by this gene is a member of the gelsolin/villin family of actin regulatory proteins. This protein has structural similarity to villin. It binds actin and may play a role in the development of neuronal cells that form ganglia. |
| B4GALT5 (Chromosome 20, 48249482..48330421, complement) | This gene is one of seven beta-1,4-galactosyltransferase (beta4GalT) genes. Each beta4GalT has a distinct function in the biosynthesis of different glycoconjugates and saccharide structures. |
| BCL2 (Chromosome 18, 60790579..60987011, complement) | This gene encodes an integral outer mitochondrial membrane protein that blocks the apoptotic death of some cells such as lymphocytes. |
| CCNJL (Chromosome 5, 159678666..159739602, complement) | cyclin-J-like protein |
| CNTNAP3 (Chromosome 9, 39070196..39288300, complement) | The protein encoded by this gene belongs to the NCP family of cell-recognition molecules. The protein encoded by this gene may play a role in cell recognition within the nervous system. |
| CREB5 (Chromosome 7, 28338940..28865511) | The product of this gene belongs to the CRE (cAMP response element)-binding protein family. |
| CSNK1E (Chromosome 22, 38686697..38714089, complement) | The protein encoded by this gene is a serine/threonine protein kinase and a member of the casein kinase I protein family, whose members have been implicated in the control of cytoplasmic and nuclear processes, including DNA replication and repair. |
| DAAM2 (Chromosome 6, 39760159..39872653) | Dishevelled associated activator of morphogenesis 2 |
| DB304731, GCA (GRCh37: Chromosome 2, 163219187..163219246)  GRCh36: chr2:162927433-162927492 | GCA, This gene product, grancalcin, is a calcium-binding protein abundant in neutrophils and macrophages |
| FAM126B Chromosome 2, 201838441..201936392, complement) | Family with sequence similarity 126, member B |
| GK (Chromosome X, 30671476..30749579) | The protein encoded by this gene belongs to the FGGY kinase family. This protein is a key enzyme in the regulation of glycerol uptake and metabolism. |
| HCG27 (Chromosome: 6, 31165537..31171745) | HLA complex group 27 (non-protein coding) |
| HNRPDL HNRNPDL (Chromosome: 4, 83343717..83351378, complement) | heterogeneous nuclear ribonucleoprotein D-like. This gene belongs to the subfamily of ubiquitously expressed heterogeneous nuclear ribonucleoproteins (hnRNPs). The hnRNPs are RNA binding proteins and they complex with heterogeneous nuclear RNA (hnRNA). These proteins are associated with pre-mRNAs in the nucleus and appear to influence pre-mRNA processing and other aspects of mRNA metabolism and transport. |
| MAL (Chromosome 2, 95691400..95719737) | The protein encoded by this gene is a highly hydrophobic integral membrane protein belonging to the MAL family of proteolipids. The protein has been localized to the endoplasmic reticulum of T-cells and is a candidate linker protein in T-cell signal transduction. In addition, this proteolipid is localized in compact myelin of cells in the nervous system and has been implicated in myelin biogenesis and/or function. The protein plays a role in the formation, stabilization and maintenance of glycosphingolipid-enriched membrane microdomains. |
| MPZL1 (Chromosome 1, 167691187..167761156) | myelin protein zero-like 1 |
| NELL2 (Chromosome 12, 44902058..45307711, complement) | The protein encoded by this gene is a glycoprotein containing several von Willebrand factor C domains and epidermal growth factor (EGF)-like domains. |
| NFAM1 (Chromosome 22, 42776413..42833086, complement) | The protein encoded by this gene is a type I membrane receptor that activates cytokine gene promoters such as the IL-13 and TNF-alpha promoters. The encoded protein contains an immunoreceptor tyrosine-based activation motif (ITAM) and is thought to regulate the signaling and development of B-cells |
| NR1D2 (Chromosome 3, 23986751..24022109) | This gene encodes a member of the nuclear hormone receptor family, specifically the NR1 subfamily of receptors. The encoded protein functions as a transcriptional repressor and may play a role in circadian rhythms and carbohydrate and lipid metabolism. |
| OTX1 (Chromosome 2, 63277192..63284966) | This gene encodes a member of the bicoid sub-family of homeodomain-containing transcription factors. The encoded protein acts as a transcription factor and may play a role in brain and sensory organ development |
| SLC22A4 (Chromosome 5, 131630145..131679899) | Polyspecific organic cation transporters in the liver, kidney, intestine, and other organs are critical for elimination of many endogenous small organic cations as well as a wide array of drugs and environmental toxins. |
| SLC6A6 (Chromosome 3, 14444076..14530857) | This gene encodes a multi-pass membrane protein that is a member of a family of sodium and chloride-ion dependent transporters. The encoded protein transports taurine and beta-alanine. |
| ST6GALNAC2 (Chromosome 17, 74561461..74582145, complement) | ST6GALNAC2 belongs to a family of sialyltransferases that add sialic acids to the nonreducing ends of glycoconjugates. At the cell surface, these modifications have roles in cell-cell and cell-substrate interactions, bacterial adhesion, and protein targeting (Samyn-Petit et al., 2000) |
| TMEM140 (Chromosome: 7, 134832766..134850967) | transmembrane protein 140 |
| TREM1 (Chromosome 6, 41242999..41254457, complement) | This gene encodes a receptor belonging to the Ig superfamily that is expressed on myeloid cells. This protein amplifies neutrophil and monocyte-mediated inflammatory responses triggered by bacterial and fungal infections by stimulating release of pro-inflammatory chemokines and cytokines, as well as increased surface expression of cell activation markers. |
